# Glaucoma: Novel antifibrotic therapeutics for the trabecular meshwork

**DOI:** 10.1016/j.ejphar.2023.175882

**Published:** 2023-09-05

**Authors:** Mengqi Qin, Cynthia Yu-Wai-Man

**Affiliations:** King's College London, London, SE1 7EH, UK

**Keywords:** Trabecular meshwork, Glaucoma, MIGS, Fibrosis

## Abstract

Glaucoma is a chronic and progressive neurodegenerative disease characterized by the loss of retinal ganglion cells and visual field defects, and currently affects around 1% of the world's population. Elevated intraocular pressure (IOP) is the best-known modifiable risk factor and a key therapeutic target in hypertensive glaucoma. The trabecular meshwork (TM) is the main site of aqueous humor outflow resistance and therefore a critical regulator of IOP. Fibrosis, a reparative process characterized by the excessive deposition of extracellular matrix components and contractile myofibroblasts, can impair TM function and contribute to the pathogenesis of primary open-angle glaucoma (POAG) as well as the failure of minimally invasive glaucoma surgery (MIGS) devices. This paper provides a detailed overview of the current anti-fibrotic therapeutics targeting the TM in glaucoma, along with their anti-fibrotic mechanisms, efficacy as well as the current research progress from pre-clinical to clinical studies.

## 1 Introduction

Glaucoma characterized by irreversible retinal ganglion cell (RGC) apoptosis is the leading cause of global blindness and affects more than 70 million patients worldwide (Weinreb et al., 2014). As glaucoma is usually asymptomatic in the early stage, about 10–50% of affected individuals are generally diagnosed only late in the disease; finding an effective therapeutic strategy for glaucoma therefore remains a high priority (Leite et al., 2011; Rotchford et al., 2003). Although there is a growing recognition that an increasing prevalence of individuals has been diagnosed with glaucoma characterized by normal intraocular pressure (IOP), abnormal elevation of IOP continues to be identified as a main risk factor responsible for the RGCs death and optic nerve damage (Adornetto et al., 2020). Consequently, for glaucoma management, the adequate reduction of IOP through the administration of hypotensive pharmacological agents or the implementation of surgical interventions remain the main therapeutic modality presently (Esporcatte and Tavares, 2016; Lusthaus and Goldberg, 2019).

Fibrosis, a tissue repair response characterized by excessive depositions of extracellular matrix (ECM) components like collagen, fibronectin (FN), fibrillin and hyaluronic acid, is a common pathological feature leading to morbidity and mortality in many chronic diseases (Gauthier and Liu, 2017; Henderson et al., 2020; O'Regan et al., 2021). The prevailing notion holds that fibrosis is a highly dynamic process secondary to various harmful agents and mechanisms followed by several sequential events: when the tissue injury occurs, the tissue macrophages are activated and produce a large variety of cytokines and chemokines which can lead to the local activation of the fibroblast, a tissue-resident mesenchymal cells contribuingd to ECM production. The fibroblasts then transition to myofibroblasts or other collagen-producing mesenchymal populations, which can increase their contractility, enhance the secretion of ECM components as well as inflammatory mediators, and initiate the wound healing response (Di Carlo and Peduto, 2018; Weiskirchen et al., 2019).

**Fig. 1:**
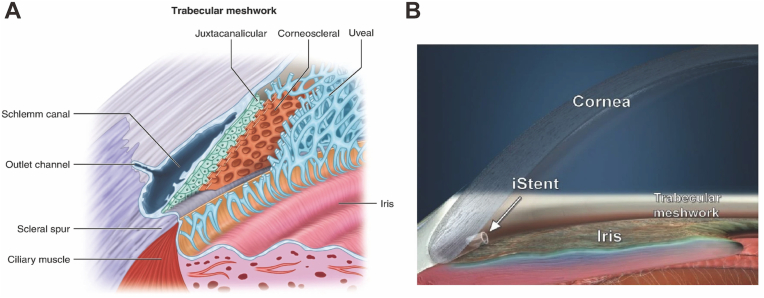
**(A)** Schematic illustration of trabecular meshwork tissue (Lam and Lawlor, 2021). **(B)** Appearance and location of a MIGS device, for example iStent inject (Paletta Guedes et al., 2022).

In the adult human eye, an estimated 80–90% of aqueous humor (AH) outflow occur through the trabecular meshwork (TM) tissue. The TM, a sieve-like tissue located in the iridocorneal angle of the anterior chamber, serves as the primary outflow pathway by working as a filter that evacuates the aqueous humor collected from the anterior chamber angle into the Schlemm's canal (Buffault et al., 2020). Anatomically, ECM is the essential component for three sections of the TM: the corneoscleral, uveoscleral, and juxtacanalicular layers, in which the trabecular cells can wrap around the ECM and form an irregular and interwoven spatial network (Fig. 1A) (Pouw et al., 2021). Severe fibrosis in the TM can lead to continuous abnormal ECM accumulation and distortion of the TM framework, and thereby result in increased resistance to AH outflow and elevated IOP. In addition, minimally invasive glaucoma surgery (MIGS) is an emerging glaucoma surgical treatment modality; these procedures can increase AH outflow by targeting the TM (Hydrus stent, Kahook dual blade, iStent, Trabectome), ciliary body (endocyclophotocoagulation) or creating the aqueous shunt in subconjunctival space (XEN implant) to achieve a rapid and effective reduction in IOP with a high safety profile (Fig. 1B) (Dhingra and Bhartiya, 2020). However, fibrosis in the TM tissue can adversely affect the implanted stents and is the main cause of failure in MIGS (Luo et al., 2022). Anti-fibrotic therapeutics targeting the TM thus represent a promising strategy in glaucoma management by reducing IOP as well as improving the surgical success rates of MIGS devices.

In recent years, various compounds with anti-fibrotic properties have been investigated as potential therapeutics for glaucoma. These compounds can target different pathways involved in fibrosis, such as transforming growth factor-beta (TGF-β) signaling, Rho kinase (ROCK) signaling, and ECM synthesis and degradation. TGF-β is a group of multifunctional cytokines that correlate with ECM deposition, and anti-TGF-β approaches appear to be a promising strategy in treating human fibrotic diseases (Peng et al., 2022; Xu et al., 2016). TGF-β2 whose level is obviously elevated in the AH and TM tissue of primary open-angle glaucoma (POAG) patients is the most abundant TGF-β isoform in the eye and has been found to closely relate to the fibrotic pathogenesis in TM (Wang et al., 2017; Wordinger et al., 2014). It has been implicated that overexpression of TGF-β2 can promote TM cells to secrete ECM crosslinking enzymes, such as tissue transglutaminase 2 (TGM2) (Wordinger et al., 2014). By triggering covalent crosslinking of ECM, TGM2 can decrease the ECM turnover, increase the tissue stiffness and finally lead to a fibrotic phenotype in the TM (Raychaudhuri et al., 2017). Several TGF-β inhibitors have been tested in preclinical studies for their potential as anti-fibrotic therapeutics in glaucoma. These include small molecule inhibitors, such as SB-431542 (Teplitsky et al., 2023) and LY-364947 (Bermudez et al., 2016), and biologics, such as anti-TGF-β2 antibody (Mead et al., 2003). ROCK is a downstream effector of the Rho GTPase family, which regulates cytoskeletal dynamics and the contractile tone of smooth muscle tissues (Amano et al., 2010). Preclinical studies have implicated the importance of ROCK signaling in the regulation of TM contractility and ECM synthesis (Buffault et al., 2022; Pitha et al., 2018). So far, several ROCK inhibitors, such as PHP-201 and AR-12286, have been proven to reduce IOP in animal models of glaucoma and are currently being evaluated in clinical trials (Ha et al., 2022; Ren et al., 2023). In addition, the potent Rho kinase/norepinephrine transporter inhibitor, netarsudil, is already commercially available for glaucoma treatment due to its hypotensive effect and favorable safety profile, as demonstrated in clinical trials (Mehran et al., 2020). ECM synthesis and degradation are tightly regulated processes that play a critical role in maintaining the structural and functional integrity of the TM. Dysregulation of these processes can lead to fibrosis and impaired TM function. Several compounds that target ECM synthesis and degradation pathways, such as decorin, lysyl oxidase-like 1 (LOXL1), and matrix metalloproteinases (MMPs), have been investigated for their potential as anti-fibrotic therapeutics in glaucoma (Nikhalashree et al., 2022; Schlotzer-Schrehardt and Zenkel, 2019; Weinreb et al., 2020).

**Fig. 2:**
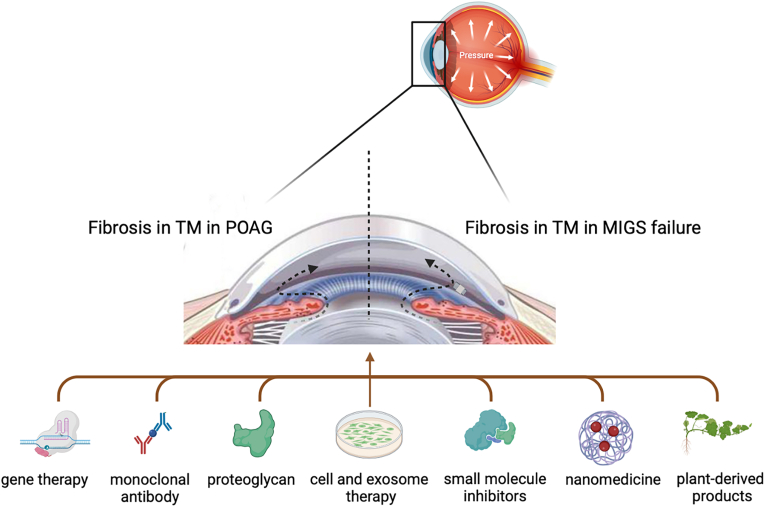
Summary of various therapeutic approaches with anti-fibrotic properties in the TM.

Whilst these therapeutics with anti-fibrotic properties show promising results, a definitive therapy for this disease has yet to be identified and a significant proportion of glaucoma patients remain refractory to treatment. Hence, there are still formulary improvements and fixed combinations that need to be addressed. As the TM constitutes the main drainage pathway for regulating the outflow of aqueous humor and is a direct target in MIGS procedures, anti-fibrotic therapeutics specifically focusing on the TM can be a promising therapeutic strategy in glaucoma, especially POAG. This review aims to provide a detailed overview of the current state of research on anti-fibrotic therapeutics by targeting the TM in glaucoma treatment. We will focus on the recent advances and anti-fibrotic effects of different types of therapeutic approaches, including gene therapy, monoclonal antibody, proteoglycan, small molecule inhibitor, plant-derived product, nanomedicine, as well as cell and exosome therapy (Fig. 2), and summarize the mechanisms underlying the anti-fibrotic effects of these therapeutics on the TM (Table 1).

**Table 1:** Efficiencies of different anti-fibrotic agents in TM cells and AH outflow pathway.

Gene therapy			
Mechanism	Agent	Cell line/tissue	Efficiency and Potency against fibrosis
**miRNA**	miR-200c	human TM cell (Luna et al., 2012)	Downregulating the expression of several genes involved in actin regulation and cell contraction, such as *ZEB1, ZEB2, ETAR, LPAR1, FHOD1, RHOA*.
		rat (Luna et al., 2012)	The rat model that received miR-200c mimic by intracameral injection showed a significant IOP reduction compared to control, especially at day 8 (21.2 mmHg vs 15.1 mmHg) with good *in vivo* tolerance.
	miR-146a	human TM cell (Li et al., 2010)	Preventing alterations of the extracellular proteolytic activity of the TM by downregulating expression of PAI1.
		rat (Luna et al., 2021)	Both lentiviral and adenoviral vectors expressing miR-146a intracameral injection can result in IOP reduction ranging from 2.6 to 4.4 mmHg, which can be sustained for at least two months. Administration of lentiviral miR-146a in the anterior chamber showed a significant effect on IOP decrease (4.4 ± 2.9 mmHg) and can last until rats were sacrificed more than 8 months later without signs of inflammation or other abnormalities.
	miR-29	human TM cell (Luna et al., 2009; Villarreal et al., 2011)	Suppressing various EMC, like SPARC, collagen I, collagen IV, and laminin expression and negatively regulate genes involved in ECM deposition and remodeling.
	miR-486–5p	human TM cell (Xu et al., 2022)	Inhibiting H_2_O_2_-stimulated ECM remodeling by blocking TGF-β2/Smad2 signaling pathway.
	miR-483–3p	human TM cell (Shen et al., 2015)	Protecting TM cell from 300 μM H_2_O_2_ induced-fibrosis by inhibiting TGF-β2/Smad4 signaling pathway.
	miR-18a-5p	human TM cell (Knox et al., 2022)	Reducing TGF-β2-mediated TM cell contractility and CTGF expression.
	miR-137	human TM cell (Wang et al., 2021)	Decreasing ECM production by blocking the YAP/TAZ signaling pathway.
	miR-24	human TM cell (Luna et al., 2011)	Targeting the subtilisin-like proprotein convertase and therefore disrupting the processing of TGF-β1 induced-fibrotic events.
	miR-1	human TM cell (Guo et al., 2019)	Decreasing mRNA and protein levels of FN directly targeting 3′-UTR.
**siRNA**	siRNA-paxillin	monkey TM cell (Takahashi et al., 2014)	Decreasing level of fibronectin and actin stress fibers induced by ECM stimulation and blocking in EMT-like alteration.
	siRNA-CTGF	human TM cell (Junglas et al., 2009, 2012)	Decreasing TM actin cytoskeleton and inhibiting TGF-β2-induced upregulation of FN.
	siRNA-Smad7	human TM cell (Su et al., 2012)	Interrupting the effects of TGF-β2 on the expression of ECM components, such as fibronectin and laminin.
	siRNA-SPARC	human TM cell (Wei et al., 2012)	Downregulating the expression of FN.
	siRNA-VLK	human TM cell (Maddala et al., 2017)	Decreasing the tyrosine phosphorylation of ECM proteins, and influencing contractile and adhesive properties.
**shRNA**	shRNA-GAS5	human TM cell (Meng et al., 2022)	Alleviating H_2_O_2_ induced ECM deposition by GAS5/miR-29b-3p/STAT3 axis.
**Antisense Oligonucleotides**	laminin ASOs	bovine trabecular meshwork cell (Tane et al., 2004, 2007)	Reversing the laminin upregulated profile and attenuating TM stiffness which is manifested by a decreased *in vitro* permeability and increased transelectrical resistance.
	collagen type IV ASOs		
	FN ASOs	glaucoma patient (Oshitari et al., 2006)	Increasing aqueous outflow facility and inhibiting ECM overexpression compared with untreated glaucomatous eye.
**Monoclonal antibody**			
**Agent**	**Target**	**Cell line/tissue**	**Efficiency and Potency against fibrosis**
bevacizumab	VEGF	human TM cell (Kahook & Ammar, 2010)	Exerting anti-metabolic and anti-proliferative effects.
		glaucoma patient (Yoshida et al., 2011)	Attenuating tissue oedema, fibrinous material deposition, and inflammatory cells infiltration.
GDF7 neutralizing antibody	GDF7	human TM cell (Wan et al., 2021)	Reversing the fibrotic phenotype, such as significant accumulation of collagen, α-SMA, and fibronectin in TM cells.
		rhesus monkey (Wan et al., 2021)	Inhibiting TM fibrosis, and improving aqueous humor outflow facility (from 0.1 to 0.3 μL/min·mmHg), effectively control the IOP (from 21.3 ± 0.3 to 17.6 ± 0.2 mmHg).
CTGF neutralizing antibody	CTGF	human TM cell (Wallace et al., 2013)	Decreasing the expression of fibrotic genes and extracellular matrix production in the TM cell under H_2_O_2_ treatment.
**Proteoglycan**			
**Agent**	**Category**	**Cell line/tissue**	**Efficiency and Potency against fibrosis**
latanoprost acid PGF2α	prostaglandin analogs	human TM cell (Anthony et al., 1998; Bahler et al., 2008; Fuchshofer et al., 2014; Oh et al., 2006)	Mediating intracellular calcium release, increasing expression level of MMPs or inducing ECM loss in TM.
PGF1		human (Dijkstra et al., 1999)	Increasing outflow facility in the human eye by 24%.
omidenepag	EP2 receptor agonist	human TM cell (Nakamura et al., 2021)	Decreasing TGF-β2-induced contraction as well as *α-SMA* and *COL1A* mRNA upregulation.
N(6)-cyclohexyladenosine	adenosine agonists	human TM cell (Husain et al., 2007; Shearer and Crosson, 2002)	Enhancing extracellular matrix turnover and stimulating secretion of MMP2.
clusterin	secretory chaperone protein	human TM cell (Soundararajan et al., 2022)	Increasing MMP2 activity and decreasing a group of pro-fibrotic proteins level to inhibit ECM expression and distribution.
TNFAIP3	anti-inflammatory signaling molecule	human TM cell (Mzyk et al., 2022)	Rescuing the fibrotic response in TM by blocking pathological TGF-β2-TLR signaling pathway.
yoda 1	piezo 1 agonist	human TM cell (Morozumi et al., 2022)	Promoting the degradation of fibronectin by increasing the expression of MMP2 and PGF2α and enhancing the arachidonic acid cascade.
		mice (Morozumi et al., 2021)	Decreasing fibronectin gene expression level, increasing *MMP2* expression level and suppressing human TM cells migration and proliferation to maintain the TM histological structure.
cathepsin B	lysosomal cysteine protease	human TM cell (Nettesheim et al., 2020)	Degradation of ECM in TM cells by modulating the TGF-β/Smad signaling and expression of PAI1.
angiogenin	ribonuclease secreted protein	mice (Jeong et al., 2020)	Reversing extracellular matrix deposition and fibrillar material density accumulation.
phosphatase and tensin homolog	dephosphorylate focal-adhesion kinase	human TM cell (Tellios et al., 2017)	Exogenous overexpression of a mutated form of PTEN with enhanced phosphatase activity prevented the TGF-β-induced collagen expression by TM cells.
decorin	leucine-rich proteoglycans	rat (Hill et al., 2015, 2016)	Reducing TGF-β-induced TM fibrosis, upregulating the expression levels of MMP2 and MMP9, and lowering TIMP2, a matrix metalloproteinase inhibitor level.
Arg-Gly-Asp motif	amino acid sequence	human TM cell (Hennig et al., 2016)	Inhibiting CTGF-mediated fibrotic events by attenuating extracellular matrix synthesis and stress fiber formation.
**Small molecule inhibitors**			
**Agent**	**Category**	**Cell line/tissue**	**Efficiency and Potency against fibrosis**
netarsudil	ROCK inhibitor	mice (G. Li et al., 2021)	Preventing TM stiffness and fibrotic disease processes to attenuate IOP elevation.
ceralasertib	ataxia telangiectasia and Rad3-related (ATR) kinase inhibitor	mice (Huang et al., 2022)	Alleviating impaired AH drainage by decreasing ECM remodeling and collagen production in the TM.
		human TM cell (Huang et al., 2022)	Inhibiting the expression of fibronectin, α-SMA, laminin subunit beta 1, collagen I, and collagen IV, and reducing altered cytoskeleton and nitric oxide production in TGF-β1-induced human TM cells via the CHK1/P53 pathway.
rapamycin	mTOR inhibitor	human TM cell (Igarashi et al., 2021)	Attenuating the upregulation of fibronectin, COL1A and α-SMA induced by TGF-β2-stimulation.
torin1			
suberoylanilide hydroxamic acid	histone deacetylase inhibitor	human TM cell (Fujimoto et al., 2021)	Inhibiting the extracellular matrix proteins expression by regulating the non-Smad pathway of TGF-β signaling.
		porcine (Fujimoto et al., 2021)	Alleviating the TGF-β2-induced decrease in outflow facility (0.510 ± 0.117 μL/min/mmHg vs 0.272 ± 0.044 μL/min/mmHg).
		rabbit (Fujimoto et al., 2021)	Reversing TGF-β2-induced IOP elevation.
losartan	selective angiotensin II type 1 receptor inhibitor	human TM cell (Choi et al., 2019)	Decreasing the expression of α-SMA, connective tissue growth factor, COL1A and fibronectin.
(R)-bromoenol lactone	calcium-independent phospholipase A2 inhibitor	human TM cell (Pattabiraman et al., 2012)	Alleviating signs of hTM fibrosis, such as a decrease in actin stress fibers as well as myosin light-chain phosphorylation.
		porcine (Pattabiraman et al., 2012)	Increasing AH outflow facility by 80% by the third hour.
AS605240	PI3K isoforms (p110γ) inhibitor	human TM cell (Parapuram et al., 2019)	Preventing TGF-β-induced collagen expression and PTEN phosphorylation in TM cells with less toxicity.
5-azacytidine	DNA methylation inhibitor	human TM cell (F. McDonnell et al., 2016)	Decreasing ECM components, COL1A expression.
CCG-203971	serum response factor/myocardin-related transcription factor complex inhibitor	human TM cell (Taiyab et al., 2019)	Reversing the dexamethasone-induced hTM stiffness and decreasing the expression of ECM composition, like α-SMA.
RU 38486	glucocorticoid receptor antagonist	human TM cell (Li et al., 2002)	Decreasing fibronectin level in ECM of the cells.
**Plant-derived products**			
**Agent**	**Dosage**	**Cell line/tissue**	**Efficiency and Potency against fibrosis**
baicalin	20 μM	human TM cell (Li et al., 2023)	Inhibiting TGF-β2-induced ECM expression by repressing MyD88/NF-κB signaling pathway.
astragaloside IV	50 and 100 μM	human TM cell (Kasetti et al., 2021)	Decreasing extracellular matrix and α-SMA deposition as well as enhancing the levels and enzymatic activities of matrix metalloproteinases.
	1 mM	mice (Kasetti et al., 2021)	Decreasing FN, collagen 1, endoplasmic reticulum stress and SMA.
tetramethylpyrazine	100 μM	human TM cell (Yu et al., 2015)	Reversing TGF-β1-induced fiber accumulation and suppressing ECM accumulation induced by TGF-β2.
cannabidiol	1 μM	porcine TM cell (Aebersold and Song, 2022)	Alleviating collagen contraction and RhoA activation in cultured porcine TM cells.
	1 μM	porcine (Aebersold and Song, 2022)	Doubling aqueous humor outflow compared with the vehicle in porcine anterior segment-perfused organ culture model.
**Nanomedicine**			
**Agent**	**Nanomaterial**	**Cell line/tissue**	**Efficiency and Potency against fibrosis**
siRNA-MRTF-B	CL4H6- LNP	human TM cell (Luo et al., 2022)	Protecting siRNA from rapid degradation, effectively delivering MRTF-B siRNA into human TM cells with no toxicity and significantly reducing contractibility of TM cells.
siRNA-CTGF	hyaluronan	porcine (Dillinger et al., 2018) human (Dillinger et al., 2018)	Deeply penetrating into the outflow region of murine by targeting CD44 and can decreasing CTGF-mediated fibrosis by reduction of CTGF to about 50% in the donor cells with high basal CTGF expression.
MMP-3	PLGA	human TM cell (Turturro et al., 2013)	The sustained release of MMP3 can result in an extended fibronectin degradation period for up to 10 days in cultured TM cells.
**Cell and exosome therapy**			
**Agent**	**Cell line/tissue**	**Efficiency and Potency against fibrosis**	
MSC therapy	human TM cell (Roubeix et al., 2015)	Inhibiting TGF-β2-dependent profibrotic phenotype.	
	rat (Roubeix et al., 2015)	Decreasing IOP level by 5.1 mmHg and reversing RGCs loss caused by cauterization of episcleral veins.	
BMSC-derived exosomes	human TM cell (Y. C. Li et al., 2021)	Increasing MMP expression and inhibiting the expression of inflammatory cytokines.	

## 2 Gene therapy

The initial concept of gene therapy aiming to correct genetic defects in host cells by introducing a foreign genetic material has been around for 50 years (Amador et al., 2022). With the arrival of approved gene therapy, such as Glybera, Imlygic and Luxturna, gene therapy has successfully demonstrated a significant clinical benefit (Gordon et al., 2019; Wirth et al., 2013). Gene therapy is also a compelling therapeutic strategy for multiple ocular diseases. The reasons lie in that the eye is an immune-privileged space and treatment with viral vectors will not trigger systemic adverse events (Gordon et al., 2019). Besides, the eye can be easily accessed by techniques for injection of genetic vectors (Hu et al., 2021). All of these benefits make the eye an ideal target organ for gene therapy of both inherited as well as acquired disorders.

### 2.1 miRNA

**Fig. 3:**
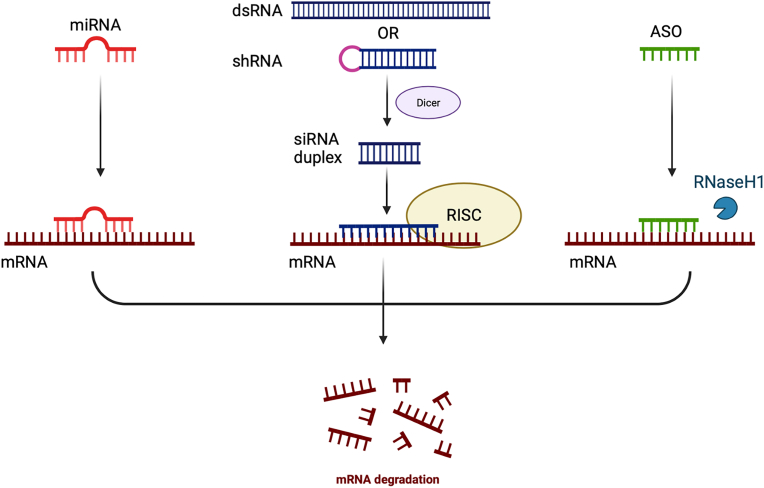
Schematic illustration of gene silencing mechanism mediated by miRNA, siRNA and ASO. miRNA, microRNA; siRNA, small interfering RNA; ASO, antisense oligonucleotide.

MicroRNA (miRNA), a short non-coding RNA fragment containing 18–22 nucleotides, can perform post-transcriptional gene regulation by influencing 3′-untranslated region (3′-UTR) in mRNA and trigger repression of translation (Hill and Tran, 2021). It has been reported that miRNA plays an important role in IOP regulation and glaucoma pathogenesis, hence the application of miRNA-based therapy holds great promise in glaucoma management (Greene et al., 2022) (Fig. 3).

MiR-200 family is a group of evolutionarily conserved miRNA (Cavallari et al., 2021) shown to possess a great clinical value for indicating prognostic and predictive information on multiple diseases, such as neurodegenerative disease (Fu et al., 2019), metastatic breast cancer (Fischer et al., 2022) and hepatocellular carcinoma (Mao et al., 2020). Previous data revealed that miR-200 is a regulator of actomyosin system and shows a potential effect on preventing the deposition of ECM and prohibiting epithelial-mesenchymal transition (Elson-Schwab et al., 2010; Patel and Noureddine, 2012). MiR-200c, a member of miR-200 family, is described to be a post-transcriptional modulator which is highly expressed on TM cells and participates in regulating cell contraction, cytoskeletal organisation and fibrogenesis (Chu et al., 2019; Li et al., 2009; Luna et al., 2012). Transfection hsa-miR-200c mimic into human trabecular meshwork (hTM) cells can regulate the tone of TM cells by directly downregulating the expression of several genes involved in actin regulation and cell contraction, such as *ZEB1, ZEB2, ETAR, LPAR1, FHOD1, RHOA*. Besides, the rat model that received miR-200c mimic by intracameral injection showed a significant IOP reduction compared to the control, especially at day 8 (21.2 mmHg vs 15.1 mmHg) with good *in vivo* tolerance (Luna et al., 2012).

MiR-146a is another miRNA target for anti-fibrotic treatment. In replicative senescent TM cells, miR-146a mimic transfection can minimize the effects of senescence on the AH outflow facility by downregulating plasminogen activator inhibitor 1 (PAI1) level, a main contributor to ECM homeostasis in the TM, and inhibiting the activity of extracellular proteinases, tissue and urokinase plasminogen activators, compared to cells transfected with scrambled control (Li et al., 2010). Besides, the animal study showed that both lentiviral and adenoviral vectors expressing miR-146a intracameral injection can result in an IOP reduction ranging from 2.6 to 4.4 mmHg, which can be sustained for at least two months. Moreover, administration of lentiviral miR-146a in the anterior chamber showed a significant effect on IOP decrease (4.4 ± 2.9 mmHg), which can last more than 8 months without signs of inflammation or other abnormalities (Luna et al., 2021). The reasons for the decrease in IOP induced by miR-146a in rats have not been verified experimentally. As miR-146a is a critical inhibitor for TGF-β, PAI1 and NF-κB signaling, the authors proposed that miR-146a can achieve these encouraging results *in vivo* by negatively regulating the fibrotic and anti-inflammatory response (Luna et al., 2021). All of those results supported that intracameral delivery of miR-146a can prevent TM fibrotic process in both normal and senescence TM cells, and provide a long-term IOP reduction in rats with a good safety profile (Luna et al., 2021).

In addition, multiple *in vitro* studies have shown that other miRNAs can protect TM fibrosis from hyperoxia stimulation and have explored the mechanisms in detail. The miR-29 family was previously shown to be an effective protectant in many diseases (Smyth et al., 2022). Besides, miR-29 was also reported to be a key modulator of ECM homeostasis. Transfection of miR-29 mimic can lead to the downregulation of various genes relevant to the synthesis and deposition of the ECM components in a chronic oxidative stress model (Cushing et al., 2015; Luna et al., 2009). Meanwhile, one of the miR-29 mimics, MRG-201, has completed Phase 2 clinical trials and confirmed its anti-fibrotic activity in the prevention or reduction of keloid formation (Clinical-Trials.gov: NCT03601052). Villarreal et al. (2011) investigated the role of all three subtypes in the miR-29 family, miR-29a, miR-29b and miR-29c, in the regulation of ECM deposition in TM cells. They found that, under TGF-β2 stimulation, the expression of miR-29a is upregulated, the miR-29b level is downregulated, and the miR-29c expression is unaffected. Despite the fact that the response to TGF-β2 stimulation is variable, the overexpression of these paralogs seems to exert a consistent effect on suppressing EMC proteins production in TM after stimulation with activated recombinant human TGF-β2 for 24 h. In addition, Luna et al. (2009) investigated the effect of miR-29b on ECM deposition in TM cells under chronic oxidative stress. They found that miR-29b can negatively regulate genes involved in ECM deposition and remodeling by directly targeting *BMP1*, *ADAM12*, and *NKIRAS2*, hence exerting a protective effect on the TM against chronic oxidative stress injury.

MiR-486–5p is a plasma and extracellular vesicle-enriched miRNA (Douvris et al., 2022). As shown by the results of small RNA sequencing, miR-486–5p was downregulated in POAG patients (Hubens et al., 2021), and transfection of miR-486–5p mimics in TM cells can significantly inhibit H_2_O_2_-stimulated ECM remodeling by blocking the TGF-β2/Smad2 signaling pathway (Xu et al., 2022). MiR-483 generally exists in two mature forms, miR-483–3p and miR-483–5p, in the body and is closely related to cell proliferation and tumor development (Watson et al., 2013; Zhou et al., 2018). Under 300 μM H_2_O_2_ stimulation, ectopic expression of miR-483–3p by lentiviral vector could protect the TM cell from fibrosis by inhibiting the TGF-β2/Smad4 signaling pathway (Shen et al., 2015). MiR-18a-5p is also an upstream regulator for TGF-β2-mediated TM cell contractility, and transfection of either synthetic miR-18a-5p mimic or the stable overexpressed miR-18a-5p lentiviral vector can both reduce the TGF-β2-mediated fibrotic response (Knox et al., 2022). Based on bioinformatic enrichment analyses, miR-137 is a predicted common gene in glaucoma, age-related neurodegenerative disease and Alzheimer's disease, and can be considered as a potential biomarker and therapeutic target in these three neurodegenerative diseases (Romano et al., 2015). To validate its role in glaucoma treatment, Wang et al. (2021) found that transfection the miR-137 mimic in hTM cells can not only attenuate cell apoptosis, but also decrease ECM production induced by oxygen stress via blocking the YAP/TAZ signaling pathway. In addition, under the cyclic mechanical stress, overexpression of miR-24 can directly target the subtilisin-like proprotein convertase and therefore disrupt the processing of TGF-β1 whose activation is associated with fibrotic events in hTM (Luna et al., 2011). Besides, miR-1 can decrease mRNA and protein levels of FN by directly targeting FN 3′-UTR to protect TM cells from fibrosis under H_2_O_2_ stimulation (Guo et al., 2019). However, further preclinical studies aimed at examination the consequence of alterations in miRNA expression on IOP level and outflow homeostasis are needed to translate miRNA-based therapy in glaucoma to clinic.

### 2.2 siRNA and shRNA

RNA interference mediated by cytoplastic double-stranded RNA is a remarkable endogenous regulatory pathway to degrade host mRNA which was first coined in 1998 by Fire and Mello (Fire et al., 1998). Small interfering RNAs (siRNAs) and short hairpin RNAs (shRNAs) are the powerful techniques for mediating RNA interference effect (Rao et al., 2009). SiRNAs are short, double-stranded RNA molecules that are typically 20–25 nucleotides in length and can lead to rapid and specific degradation of target mRNA (Alshaer et al., 2021). ShRNAs are longer, hairpin-shaped RNA molecules that can be detected by the cellular RNAi machinery and then engineered to form active siRNAs, after which gene silencing event will be initiated (Lambeth and Smith, 2013) (Fig. 3).

Excessive ECM deposition in the TM can increase outflow resistance. Paxillin is a multifunctional protein that responds to multiple ECM ligands, such as collagen I and FN, and extracellular soluble agonist, such as TGF-β1(Hagel et al., 2002). Takahashi et al. (2014) found that cultured monkey TM cells, which were transfected with siRNA-targeted paxillin, exhibited a decreased level of FN and actin stress fibers induced by ECM stimulation and a block in epithelial-to-mesenchymal transition (EMT)-like alteration.

Connective tissue growth factor (CTGF) is a crucial target molecule downstream of TGF-β. Increasing evidence have shown that CTGF is positively correlated with the degree of TM fibrosis by enhancing the ECM production (Iyer et al., 2012; Junglas et al., 2012; Su et al., 2013). *In vitro*, the overexpression of CTGF can lead to TM cell fibrosis manifested by TM contractility and ECM deposition (Junglas et al., 2012; Su et al., 2013). *In vivo*, CTGF overexpression can lead to IOP elevation, optic nerve damage, decreased aqueous humor outflow efficacy, and increased EMC deposition in the TM (Su et al., 2013). All of those results indicated that CTGF can be considered as a novel target for the treatment of POAG by regulating aqueous humor outflow resistance. By transfection of siRNA-CTGF, depletion of CTGF can lead to a significant decrease in TM actin cytoskeleton (Junglas et al., 2012) and inhibit TGF-β2-induced upregulation of FN (Junglas et al., 2009).

**Fig. 4:**
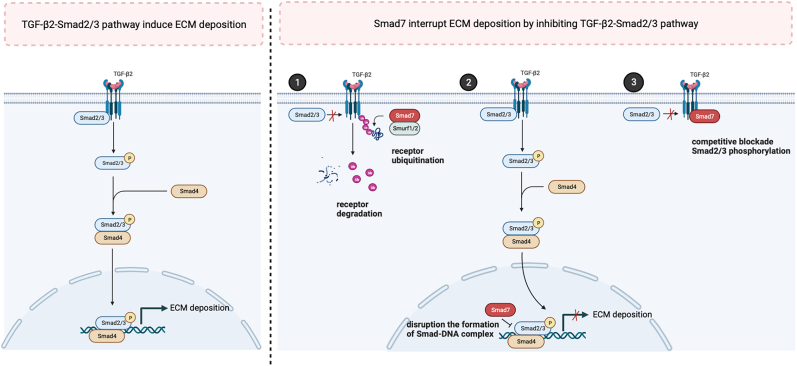
Schematic illustration of the mechanisms by which Smad7 intervenes in the deposition of extracellular matrix (ECM) through the inhibition of the TGF-β2/Smad2/3 pathway. TGF-β2, transforming growth factor-beta 2; Smad2/3, signaling against decapentaplegic 2/3; Smurf1/2, Smad ubiquitin regulatory factor 1/2; Smad4, signaling against decapentaplegic 4; Smad7, signaling against decapentaplegic 7; ECM, extracellular matrix.

TGF-β/signaling against decapentaplegic (Smad) pathway is another important axis in the regulation of ECM deposition in the TM. Smad7 is a key molecular switch that can inhibit TGF-β2/Smad2/3-mediated ECM deposition by competitively blocking phosphorylation of Smad2/3, inducing TGF-β receptor degradation and disrupting functional Smad2/3-DNA complex formation (Yan et al., 2009; Zhang et al., 2007) (Fig. 4). Despite the evidence suggesting the inhibitory effect of Smad7 on TGF-β signaling in TM cells (Fuchshofer et al., 2009; Tabak et al., 2021, Tabak et al., 2021), Su et al. (2012) found that in hTM cells, transfection with pSup-Smad7 siRNA can interrupt the effects of TGF-β2 on the production of ECM components, such as fibronectin and laminin. Although the specific mechanisms underpinning these observations remain undescribed, manipulating the expression of Smad7 can represent a potential therapeutic strategy for regulating ECM remodeling in the TM and managing POAG.

The secreted protein acidic and rich in cysteine (SPARC) is also a crucial protein which is widely distributed in TM cells and exhibits a positive relationship with collagen synthesis, secretion and ECM remodeling (Fu et al., 2021; Trombetta-Esilva and Bradshaw, 2012). Wei et al. (2012) indicated that transfection the siRNA-SPARC in TM can downregulate the expression of FN.

The secretory vertebrate lonesome kinase (VLK), which is detectable throughout the AH outflow pathway, can also alter ECM production and is suggested to play a potential role in homeostasis of the AH outflow. In hTM cells, external stimulus, such as cyclic mechanical stretch, dexamethasone and TGF-β2, can result in VLK secretion, while downregulation of VLK expression in TM cells by siRNA can decrease tyrosine phosphorylation of ECM proteins induced by TGF-β2 and influence contractile and adhesive properties (Maddala et al., 2017).

Long noncoding RNA growth arrest-specific transcript 5 (GAS5) is the upstream regulator of TGF-mediated signaling pathway (Zhang et al., 2021). In a translimbal laser photocoagulation-induced chronic glaucoma rat model, shRNA targeted for GAS5 could help to relieve glaucoma symptoms, which are manifested by reduced RGC apoptosis (Zhou et al., 2019). To study the therapeutic effect of shGAS5 on glaucoma treatment, Meng et al. (2022) transfected shGAS5 into hTM cells and found that shGAS5 effectively decreased H_2_O_2_-induced ECM deposition by the GAS5/miR-29b-3p/STAT3 axis.

### 2.3 Antisense oligonucleotides

Antisense oligonucleotides (ASOs) are short single-stranded nucleotide molecules that are designed complementarily to the targeted mRNA, and the targeted mRNA bound by ASOs will be degraded, leading to a halt in translation. Due to its superior target specificity and stability, ASOs can be a practical tool to correct genetic disorders in ocular diseases (Amador et al., 2022; Rossi et al., 2015) (Fig. 3).

Tane et al. (2004) found that the laminin expression level is increased when bovine TM cells are treated with high glucose medium or dexamethasone medium for 7 days. Transfection with laminin or collagen type IV antisense phosphorothioate oligonucleotides not only reversed the laminin upregulated profile, but also showed attenuated TM stiffness, which is manifested by a decreased *in vitro* permeability and increased transelectrical resistance. All of those results demonstrated that the ASOs strategy is a promising gene therapeutic strategy to reduce ECM deposition in TM cells.

FN ASOs have also been indicated to play an important role in maintaining AH outflow homeostasis by targeting TM tissues. Perfusing anterior chambers of glaucomatous eyes with FN ASOs for 48 h can lead to increased AH outflow facility (129 ± 3% of baseline value). FN immunostaining of TM in glaucomatous eyes was significantly increased by 160.2 ± 43.2% compared to those of normal eyes. However, histological analyses showed that there was no significant increase in the expression of ECM components, collagen IV and laminin, in TM of glaucomatous eyes perfused with FN ASOs, compared with normal eyes (Oshitari et al., 2006).

## 3 Monoclonal antibody

Bevacizumab is a well-known humanized monoclonal antibody that can bind with and inhibit the biological activities of all isoforms of vascular endothelial growth factor (VEGF). It was approved by the FDA in 2004 as an intravenous injection for certain types of cancers and can be used off-label for several eye diseases (Gunther and Altaweel, 2009; Jose et al., 2019; Yoshida et al., 2011). Yoshida et al. (2011) conducted a retrospective study in neovascular glaucoma patients who underwent trabeculectomy injected with and without 1.25 mg intravitreal bevacizumab, and revealed that, compared to the TM specimens treated with intravitreal bevacizumab, those without intravitreal bevacizumab showed more tissue oedema, deposition of fibrinous material, and infiltration of inflammatory cells. An *in vitro* experiment indicated that high doses of bevacizumab (4 mg/mL) on TM cells exerted anti-metabolic and anti-proliferative effects, and prevented the decrease in outflow pathway facility induced by TM cell metabolism and viability changes (Kahook & Ammar, 2010).

Nevertheless, several case reports have published that patients may experience sustained spikes in IOP after intravitreal injection of bevacizumab (Lanzl and Kotliar, 2017). This phenomenon could arise for several reasons. First, bevacizumab, as a relatively large 149 kDa molecule, can obstruct the AH outflow tract and lead to secondary IOP elevation (Sniegowski et al., 2010). Second, bevacizumab might exert a pro-fibrotic effect by upregulating the mRNA and protein expressions of fibrosis-related cytokines, such as CTGF and TGF-β2, in the vascular endothelial cells. The deposition of bevacizumab on the aqueous outflow channel can also induce vascular endothelial cell fibrotic process and therefore increase AH outflow resistance (Zhang et al., 2015). To further investigate the effect of bevacizumab on TM cells and AH outflow facility, some clinical trials studied the IOP lowering effect of bevacizumab in neovascular glaucoma, however, tangible clinical results are yet to be reported (Table 2).

**Table 2:** Clinical trials of new anti-fibrotic medications for glaucoma treatment.

Drug	Disease or condition targeted	Phase	Intervention	Recruitment status	ActualEnrollment	NCT
bevacizumab	neovascular glaucoma	without FDA-defined Phases	other: subconjunctival normal saline drug: bevacizumab	completed	26 participants	NCT00384631
bevacizumab	neovascular glaucoma	without FDA-defined Phases	procedure: Intracameral injection procedure: Intravitreal injection	recruiting	70 participants	NCT03648814
trabodenoson	OHT POAG	Phase 1/2	drug: trabodenoson drug: placebo	completed	144 participants	NCT01123785
trabodenoson	OHT POAG	Phase 2	drug: trabodenoson drug: latanoprost drug: timolol	completed	101 participants	NCT01917383
trabodenoson	OHT POAG	Phase 3	drug: trabodenoson 4.5% BID drug: trabodenoson 6.0% QD drug: trabodenoson 3.0% QD drug: timolol 0.5% BID drug: placebo BID	completed	303 participants	NCT02565173
cannabis	chronic medical conditions including glaucoma	Phase 2	drug: cannabis medical Device: RYAH-Medtech Inhaler	recruiting	200000 participants	NCT03944447

Growth differentiation factor 7 (GDF7) is a member of the bone morphogenic protein (BMP) family (Morotome et al., 1998). The overexpression of GDF7 is a major contributor in TM fibrosis by upregulating BMP receptor type 2/Smad signaling pathway and can result in clinical IOP elevation. The GDF7 neutralizing antibody treatment can reverse the fibrotic phenotype, such as significant accumulation of collagen, smooth muscle actin, and fibronectin in TM cells induced by GDF7 overexpression. Besides, the results of an *in vivo* experiment also supported that GDF7 neutralization effectively inhibits TM fibrosis, and delivery of GDF7 antibody into the anterior chamber in the rhesus monkey model can improve AH outflow facility (from 0.1 to 0.3 μL/min·mmHg), effectively control IOP levels (from 21.3 ± 0.3 to 17.6 ± 0.2 mmHg), and finally protect nerve fibers (Wan et al., 2021).

As a major mediator of pathological changes associated with dysfunctional AH drainage pathway, an antibody specific to CTGF emerges as a new drug delivery approach in glaucoma treatment. Humanized monoclonal anti-CTGF antibody (10 μg/mL) treatment has been shown to significantly decrease the expression of fibrotic genes and ECM production in the TM cell under H_2_O_2_ treatment (Wallace et al., 2013). Clinically, although a Phase 2, open-label, single arm clinical trial has confirmed the safety and tolerability of pamrevlumab, a well-known monoclonal antibody to CTGF, in non-ambulatory participants with Duchenne muscular dystrophy patients (Clinical-Trials.gov: NCT02606136), further research is needed to evaluate the safety and efficacy of CTGF monoclonal antibody, like pamrevlumab, for IOP control in glaucoma patients.

## 4 Proteoglycan

Prostaglandin analogs (PGAs), such as travoprost (0.004%), latanoprost (0.005%) and bimatoprost (0.03%), are first-line therapies used in the medical management of glaucoma (Tang et al., 2019). These PGAs act as prodrugs of prostaglandin F2α (PGF2α) and can enhance AH outflow facility mainly by targeting the prostaglandin F (FP) and prostaglandin E (EP) receptors located on the ciliary body (Lusthaus and Goldberg, 2016). At a molecular level, activation of FP and EP receptors in the ciliary muscle can lead to ciliary body relaxation and extracellular matrix degradation, therefore increasing the unconventional (uveoscleral) outflow (Lusthaus and Goldberg, 2016). Besides, the improvement of AH outflow by PGAs can also be achieved by influencing the conventional (TM) outflow pathway. Experimental models have provided evidence demonstrating that the administration of PGAs, such as bimatoprost and latanoprost, can decrease TM stiffness by mediating intracellular calcium release, increasing MMP expression or inducing ECM loss within the TM (Winkler and Fautsch, 2014).

Furthermore, glaucoma patients treated with PGAs and β-blockers combination can significantly reduce pro-fibrotic gene expression in the TM, which is achieved by the Smad-dependent signaling pathway (Machiraju et al., 2019; Tejwani et al., 2020). In addition to the PGAs, the prostaglandin E2 (EP2) receptor agonist also can target the TM and modulate conventional AH outflow pathways. Omidenepag (OMD) is a novel selective EP2 receptor agonist and OMD treatment for 24 h significantly decreased TGF-β2-induced contraction, as well as α-smooth muscle actin (α-SMA) and collagen 1A (COL1A) mRNA upregulation, in TM cells (Nakamura et al., 2021). Omlonti (omidenepag isopropyl ophthalmic solution 0.002%), whose active ingredient can be hydrolyzed to omidenepag during corneal penetration (Duggan, 2018), has successfully undergone clinical trials and has been approved by the FDA in 2022 to reduce elevated IOP in patients with open-angle glaucoma or ocular hypertension.

The IOP-lowering effects of adenosine agonists have been confirmed in many animal models, like mice, rabbits and monkeys, by increasing outflow facility. The *in vitro* results demonstrated that its effect is achieved by enhancing ECM turnover and by stimulating the secretion of MMP2 in TM (Husain et al., 2007; Shearer and Crosson, 2002). Trabodenoson, formerly designated as INO-8875, is the only adenosine agonist agent that has moved into clinical trials to assess its efficacy, tolerability, and safety in glaucoma patients (Table 2). Both the Phase 2 dose-escalation clinical trial (ClinicalTrials.gov Identifier: NCT01123785) and Phase 3 trabodenoson monotherapy study (ClinicalTrials.gov Identifier: NCT02565173) showed that high dose of trabodenoson (500 μg or 1.5% BID) exhibited an effective IOP reduction especially in the patients with an IOP baseline above 25 mmHg. The Phase 2 combination study (ClinicalTrials.gov Identifier: NCT02565173) revealed that, in adults with POAG or ocular hypertension, timolol twice daily added to latanoprost performed better IOP reduction effects compared to trabodenoson twice daily added to latanoprost (7.6 mmHg vs 5.8 mmHg) (Qiu, 2021). All of the clinical trials supported the clinical efficacy of trabodenoson eye drops in IOP maintenance.

Clusterin is a stress-activated, ATP-independent secretory chaperone protein that has been regarded as a therapeutic target in many diseases (Wilson and Zoubeidi, 2017). As the first recognized extracellular mammalian chaperone, it maintains protein homeostasis by binding to and inhibiting the aggregation of misfolding proteins (Wilson et al., 2022). Clusterin can also be considered as a therapeutic target for IOP control by modulating TM fibrotic processes. Compared with the wild type, the mice with a heterozygous deletion of clusterin exhibited a higher IOP (16.08 ± 0.21 mmHg vs 14.85 ± 0.19 mmHg) by 50 days post-birth. The decreased level of clusterin in TM cells led to a loss of actin polymerization and increased cell-ECM interactions, while constitutive expression clusterin in TM cells can significantly increase MMP2 activity and decrease the level of pro-fibrotic proteins to inhibit ECM expression and distribution (Soundararajan et al., 2022).

Tumor necrosis factor alpha-induced protein 3 (TNFAIP3) is an anti-inflammatory signaling molecule that regulates NF-κB signaling negatively and has been considered as a new druggable target that is strongly related to the fibrotic manifestations across multiple ethnic cohorts (Momtazi et al., 2019). By performing *in vitro* experiments, Mzyk et al. (2022) have indicated that overexpression of TNFAIP3 can rescue the fibrotic response in TM by blocking the pathological TGF- β2-TLR signaling pathway.

Piezo is a mechanosensitive ion channel that can transduce different mechanical stimulations into electrochemical signals (Tang et al., 2022). Ocular administration of Yoda 1, a piezo 1 agonist, for three days could reduce the IOP by 1.33 mmHg in mice. Besides, piezo 1 activation can promote the degradation of fibronectin in hTM by increasing the expression of MMP2 and secretion of PGF2α, as well as by enhancing the arachidonic acid cascade. On the other hand, piezo 1 activation can also maintain the TM histological structure by suppressing hTM cells migration and proliferation (Morozumi et al., 2021, 2022).

Moreover, cathepsin B (CTSB) is a lysosomal cysteine protease of the papain family highly bounded on the TM membrane (Mort and Buttle, 1997; Porter et al., 2013). Nettesheim et al. (2020) found that the presence of CTSB can result in pericellular degradation of ECM in TM cells, which is achieved by modulating the TGF-β/Smad signaling and expression of PAI1. Therefore, enhancing CTSB activity can be considered as a novel therapeutic target to alleviate ECM deposition and fibrosis in glaucomatous AH outflow pathway.

Angiogenin (ANG) is a secreted protein member of the ribonuclease superfamily involved in several diseases by exerting angiogenetic effects (Cucci et al., 2021; Gao and Xu, 2008). Moreover, ANG is highly concentrated in the tear fluid to preserve the normal ocular surface condition (Sack et al., 2005). By molecular farming technology, a cost-effective plant-derived ANG fusion protein (ANG-FcK) was developed and administration of the ANG-FcK in the benzalkonium chloride-induced TM degenerative mice model can alleviate IOP elevation at 3 weeks (11.6 ± 0.4 mmHg vs 15.7 ± 1.7 mmHg). By comparing phenotypic and ultrastructural changes between benzalkonium chloride-treated and ANG-benzalkonium chloride co-treated eyes, it has been found that ANG administration can reverse extracellular matrix deposition and fibrillar material density accumulation, and these results indicated that the protective effect of ANG might be related to an anti-fibrotic mechanism (Jeong et al., 2020).

Phosphatase and tensin homolog (PTEN) is a dephosphorylate focal-adhesion kinase with a major regulatory effect on ECM deposition by regulating TGF-β/Smad signaling axis. Besides, TGF-β can inhibit the PTEN activity by phosphorylating the PTEN at residues Ser380/Thr382/383 (Tellios et al., 2017). Exogenous overexpression of a mutated form of PTEN with enhanced phosphatase activity prevented the TGF-β-induced collagen expression in TM cells (Tellios et al., 2017).

Decorin is a group of small leucine-rich proteoglycans that can act as a regulator of matrix assembly and have anti-fibrotic properties. Preclinical results have shown that increasing decorin expression by gene therapy or administration can potentially serve as an excellent therapeutic approach for modifying eye diseases related to fibrosis and angiogenesis (Kubo et al., 2022). Intracameral injection of decorin could reduce TGF-β-induced TM fibrosis, upregulate the expression levels of MMP2 and MMP9, and lower tissue inhibitor of metalloproteinase 2 (TIMP2), a matrix metalloproteinase inhibitor, level. Besides, using a glaucomatous rodent model, intracameral injection of decorin for 10 days could attenuate IOP elevation (from 16 ± 1 to 10 ± 0.6 mmHg) and ultimately prevent progressive RGC loss (Hill et al., 2015, 2016). All of those preclinical observations showed that intracameral decorin injection can reverse established TM fibrosis and control IOP to a normal level, hence protecting RGC from progressive IOP-related death. A Phase 1 clinical trial that was launched in 2019 aimed to compare the antifibrotic effect of decorin in sub-scleral trabeculectomy versus mitomycin-C, but the results have not yet been published (Clinical-Trials.gov: NCT03924544).

Arg-Gly-Asp (RGD) motif possesses peptide antagonizing α_v_β_3_ and α_v_β_5_ integrins, which are widely expressed by TM cells and can trigger CTGF-induced fibrosis in TM by binding to CTGF directly, making it a suitable candidate to inhibit fibrosis in TM. Hennig et al. (2016) verified that cyclic RGD peptides can show specific receptor-mediated uptake into TM, and significantly inhibit CTGF-mediated fibrotic events by attenuating ECM synthesis and stress fiber formation after incubation with hTM cells.

## 5 Small molecule inhibitors

ROCK is a serine/threonine kinase that plays an important role in regulating numerous pro-stiffening and pro-fibrotic signaling pathways in many cell types (Yu et al., 2020). Rhopressa is a ROCK inhibitor that has received FDA approval to treat glaucoma and ocular hypertension in 2017. In a retrospective study, G. Li et al., 2021, Li et al., 2021 found that netarsudil, the main active ingredient in rhopressa, can lead to a clinical lowering of IOP within one month of treatment in glucocorticoid-induced glaucoma patients whose IOPs were not adequately controlled by standard anti-glaucoma medications, such as prostaglandin analogs. The results from the glucocorticoid-induced ocular hypertension mouse model showed that, by directly acting on conventional outflow cells, netarsudil can reduce and prevent TM stiffness and fibrotic processes to attenuate IOP elevation. Besides, in rabbit and monkey models, netarsudil can produce large IOP reductions which can be sustained for at least 24 h after a once daily dosing, with favorable ocular tolerability (Lin et al., 2018).

Ceralasertib, also known as AZD6738, is an ataxia telangiectasia and Rad3-related (ATR) kinase inhibitor that can inhibit the fibrotic response in the TM and decrease IOP. In the TGF-β2-induced high IOP mice model, AZD6738 could reverse IOP elevation either at an early stage or when the high IOP had already occurred. Besides, AZD6738 can alleviate impaired AH drainage by decreasing ECM remodeling and collagen production in the TM. Mechanically, AZD6738 can inhibit the expression of fibronectin, α-SMA, laminin subunit beta 1, collagen I, and collagen IV, and reduce altered cytoskeleton and nitric oxide production in hTM cells via the CHK1/P53 pathway. These results demonstrate that AZD6738 is a novel potential therapeutic modality in glaucoma (Huang et al., 2022).

Mammalian target of rapamycin (mTOR) is a protein kinase which can act as a TGF-β2 downstream factor to induce a cascade of bioactive alterations, such as fibrosis. The mammalian target of rapamycin complex (mTORC) inhibitors have been shown to attenuate fibrosis in various experimental models, suggesting that targeting mTOR signaling may be a promising therapeutic target for the treatment of fibrotic diseases (Geissler and Schlitt, 2010; Lawrence and Nho, 2018). Pretreating hTM cells with 100 nM rapamycin or torin1, two mTOR inhibitors, for 30 min can significantly attenuate the upregulation of fibronectin, COL1A and α-SMA induced by TGF-β2-stimulation (Igarashi et al., 2021).

Histone deacetylase (HDAC) is a group of evolutionarily conserved enzymes that catalyse the removal of acetyl groups from nucleosomal histones. Numerous studies have established the functional importance of specific types of HDACs in different forms of fibrosis and demonstrated the efficacy of HDAC inhibitors in ameliorating the disease in animal models (Yoon et al., 2019). Suberoylanilide hydroxamic acid (SAHA) is the first HDAC inhibitor to receive FDA approval as an anti-cancer drug (Marks and Breslow, 2007). To specify the SAHA therapeutic effect and mechanisms in glaucoma treatment, Fujimoto et al. (2021) perfused enucleated porcine eyes with 10 ng/mL recombinant porcine TGF-β2 with or without 5 μM SAHA for 72 h, and the results showed that TGF-β2 and SAHA simultaneous perfusion can significantly suppress the TGF-β2-induced decrease in AH outflow facility. SAHA coadministration can also reverse TGF-β2-induced IOP elevation in rabbits. Furthermore, the results obtained *in vitro* indicated that SAHA inhibited the activating effects of TGF-β2 on ECM proteins by regulating the non-Smad pathway of TGF-β signaling in hTM cells.

Losartan is a selective angiotensin II type 1 receptor (AT1R) inhibitor that can block TGF-β1 action and has gained interest as a potential target to reduce fibrosis. In TM cells treated with TGF-β1, losartan treatment for 48 h can markedly decrease the expression of α-SMA, connective tissue growth factor, COL1A and fibronectin, compared with cells treated with TGF-β1 alone (Choi et al., 2019).

Calcium-independent phospholipase A2 (iPLA2), a fatty acid hydrolase, is widely localized in the AH outflow pathway, including the TM in humans. IPLAs as well as its lipid products play crucial roles in diverse cellular responses, including cytoskeletal changes and tissue contraction signal transduction. Theoretically, according to their distribution in the eye and their role in diverse cellular activities, iPLAs are predicted to modulate the AH outflow facility by influencing the contractile property of the cell. Incubating hTM with an iPLA2 specific inhibitor, (R)-bromoenol lactone (R-BEL), can alleviate signs of hTM fibrosis, such as a decrease in actin stress fibers as well as myosin light-chain phosphorylation. Furthermore, perfusion with R-BEL for 3 h can increase AH outflow facility by 80% in enucleated porcine eyes (Pattabiraman et al., 2012).

Phosphatidylinositol-3-kinase (PI3K) is a key downstream player of TGF-β-mediated fibrosis (Pozzi, 2012). In TM cells, inhibiting PI3-kinase signaling can prevent the PTEN activity suppression induced by TGF-β (Tellios et al., 2017). Parapuram et al. (2019) found that only AS605240, the inhibitor specific to certain PI3-kinase isoforms (p110γ), could effectively prevent TGF-β-induced collagen expression and PTEN phosphorylation in TM cells with less toxicity, and could be used as an anti-fibrotic agent targeting the TM tissue in glaucoma patients.

DNA methylation induced by exogenous stress, such as hypoxia, is an epigenetic mechanism and an upstream regulator driving the fibrotic process through TGF-β1 and Ras protein activator like 1 (Bechtel et al., 2010). An elevated DNA methylation level can be found in lamina cribrosa cells (F. S. McDonnell et al., 2016, McDonnell et al., 2016), Schlemm's canal endothelial cells (Cai et al., 2020), and TM cells (F. McDonnell et al., 2016) from the glaucomatous patients. 5-Azacytidine (5-aza) is a methylation inhibitor. In glaucomatous hTM cells, 5-aza treatment can decrease DNA methylation level and TGF-β1 expression level, leading to a corresponding decrease in COL1A expression level (F. McDonnell et al., 2016). The results demonstrated that 5-aza can be a potential candidate to exert an anti-fibrotic effect in TM cells by modulating DNA methylation.

Serum response factor/myocardin-related transcription factor (SRF/MRTF) complex has been shown to be responsible for pathological fibrosis in many organs, such as the eye (Tagalakis et al., 2018), colon (Johnson et al., 2014), kidney (Miranda et al., 2021) and lung (Pawelec et al., 2022). CCG-203971 is a novel inhibitor of the SRF/MRTF complex and can work as potential antifibrotic therapeutics to prevent scar tissue formation in rabbit eyes after glaucoma filtration surgery (Yu-Wai-Man et al., 2017). Incubating hTM cells with CCG-203971 (5 μM) for 72 h can significantly reverse the dexamethasone-induced hTM stiffness and decrease the expression of ECM composition, such as α-SMA (Taiyab et al., 2019).

Moreover, RU 38486 is a glucocorticoid receptor antagonist. Following incubation with dexamethasone, treatment of hTM with RU 38486 can decrease fibronectin level in the hTM cells and hence exert a good anti-fibrotic effect (Li et al., 2002).

## 6 Plant-derived products

Baicalin, the major bioactive compound isolated from *Scutellaria radix*, has been explored to possess multiple biological effects, such as anti-inflammatory, antioxidant, anticancer and antimicrobial properties (Ganguly et al., 2022). This pharmacological agent can inhibit TGF-β2-induced ECM expression by repressing MyD88/NF-κB signaling pathway in hTM cell (Li et al., 2023), showing that baicalin can be regarded as a potential candidate for glaucoma treatment.

Astragaloside IV, a cycloartane-type triterpene glycoside chemical, is the major component in *Astragalus membranaceus* (Zhang et al., 2020). Astragaloside IV can exert antifibrotic effects in TGF-β2-treated TM cells by decreasing ECM deposition as well as enhancing the levels and enzymatic activities of MMPs. Furthermore, the efficacy of astragaloside IV eye drops in regulating AH outflow has been confirmed in an ocular hypertension mice model, with both histological evidence and IOP results. It has been demonstrated that the mice that received 1 mM astragaloside IV eye drops twice a day exhibited a decrease in ECM accumulation in TM tissues, as well as a significant reduction in IOP levels compared with the vehicle-treated control (16.58 ± 0.594 mmHg vs 20.5 ± 0.436 mmHg) (Kasetti et al., 2021).

Tetramethylpyrazine (TMP) is the active ingredient of traditional Chinese herb Chuanxiong and has been used in the medical treatment of glaucoma (Yu et al., 2015). In primary human TM cells, TMP can reverse TGF-β1-induced fiber accumulation and suppress ECM deposition induced by TGF-β2 (Yu et al., 2015). Furthermore, cannabidiol (CBD) whose medical value was recognized by the United Nations (UN) in 2020 has been found to alleviate collagen contraction and RhoA activation in cultured porcine TM cells (Bilbao and Spanagel, 2022). Additionally, it was suggested that 1 μM CBD can double AH outflow compared with the vehicle in porcine anterior segment-perfused organ culture model (Aebersold and Song, 2022). Currently, CBD has come into clinical trial as cannabis to determine its safety and efficacy for a wide range of chronic diseases, such as glaucoma, cancer and multiple sclerosis (ClinicalTrials.gov Identifier: NCT03944447) (Table 2).

## 7 Nanomedicine

Viral vectors are efficient delivery agents but their immunogenicity limit their clinical use. Nanoparticles conjugated with therapeutic agents, such as siRNA, miRNA and antibody, can minimize cellular toxicity and hence have been a growing area of interest. The SRF/MRTF pathway is a major cytoskeletal regulator and represents a promising anti-fibrotic target. By utilising novel lipid nanoparticles (LNP), Luo et al. (2022) synthesized PEGylated CL4H6-MRTF-B siRNA-loaded LNPs, which can protect siRNA from rapid degradation, with the aim of preventing TM fibrosis after MIGS. The results suggested that the novel CL4H6-LNPs can effectively and safely deliver MRTF-B siRNA into hTM cells, and can significantly reduce the contractibility of TM cells after transfection.

CD44 cell-surface receptor, expressed on TM and Schlemm's canal cells under normal conditions, can be upregulated in the TM cells of glaucoma patients (Dillinger et al., 2018). To increase the therapeutic efficacy of this anti-fibrotic gene therapy, Dillinger et al. (2018) developed hyaluronan-coated poly(lactic-*co*-glycolic acid)-poly(ethylene imine) nanoparticles encapsulated with siRNA-CTGF that can deeply penetrate into the outflow region of murine, porcine as well as human eyes *ex vivo* by targeting CD44 on TM and Schlemm's canal cells. It has demonstrated that these nanoparticles can decrease CTGF-mediated fibrosis in the TM by reducing CTGF levels to about 50% in donor cells with high basal CTGF expression.

In order to achieve a slow and sustained release profile of active MMP3 in cultured hTM cells, Turturro et al. (2013) also encapsulated MMP3 into poly(lactic-*co*-glycolic acid) (PLGA) microparticles with an encapsulation efficiency of about 50%, and the sustained release of MMP3 can result in an extended fibronectin degradation period for up to ten days in cultured TM cells.

## 8 Cell and exosome therapy

Mesenchymal stem cells (MSCs) represent a group of adult pluripotent stem cell that can be found in all tissues. Due to its capacity to differentiate into several cell types, MSC therapy has been recognized as a promising strategy to treat various degenerative diseases. Besides, MSC can secrete multiple trophic and immunomodulatory cytokines that are beneficial to other cells (Meirelles Lda et al., 2009). In an ocular hypertension model induced by the cauterization of three episcleral veins (ECV), Roubeix et al. (2015) injected MSCs which are isolated from rat bone marrow into the anterior chamber for two days. This procedure led to a decrease in IOP by 5.1 mmHg and reversed RGCs loss caused by ECV. With the aim to further decipher the underlying mechanism of MSCs therapy on IOP lowering, the authors tested the effect of MSCs on primary hTM cells and showed that MSCs not only inhibited the TGF-β2-dependent profibrotic phenotype acquisition in hTM, but also protected hTM from benzalkonium chloride toxicity by activating anti-apoptotic pathways.

The exosomes derived from bone marrow MSCs can also exhibit remarkable therapeutic effect to inhibit TM fibrosis. Exosomes range from 40 to 150 nm in size and are phospholipid bilayer extracellular vesicles comprised of nucleotides, lipids and proteins (Tabak et al., 2021). Exosomes can participate in intracellular communication and regulate physiological process by fusion to the target cell and releasing their cargo or binding to the receptor on the recipient cells, thereby triggering a signaling pathway (He et al., 2018). Y. C. Li et al. (2021) have found that the hTM cells which were pretreated with BMSC-derived exosomes for 24 h could reverse TM cell dysfunction by increasing MMP expression and inhibiting the expression of inflammatory cytokines.

## 9 Discussion and future perspectives

Glaucoma is a leading cause of blindness worldwide and elevated IOP is a major risk factor for the disease. As the main drainage pathway for regulating the outflow of AH and a direct target for MIGS procedures, fibrosis in the TM not only can contribute to TM stiffness and the increased resistance in AH outflow facility in glaucoma but also directly affects the surgical outcomes in MIGS. Therefore, anti-fibrotic therapeutics targeting the TM can represent a promising strategy for reducing IOP and for improving the surgical outcomes in glaucoma.

In this paper, we examined the therapeutic agents that have shown promise for alleviating fibrogenesis on the TM. These treatments discussed here complement the established glaucoma therapeutic modality. However, it is important to acknowledge that the AH outflow resistance elevation is caused by a complex pathophysiological process. Apart from TM fibrosis, there are various mechanisms eliciting key roles in its pathogenesis, such as trabecular cell senescence and apoptosis (Buffault et al., 2020). Hence, in the future, the focus will move beyond merely anti-fibrotic control and more potential molecules contributing to the etiology of glaucoma will need further exploration.

Besides, apart from the prostaglandin analogs, rhopressa and omlonti that are already commercially available, most of the anti-fibrotic compounds discussed here are still in the preclinical or early clinical stages, and could add to the armamentarium of treatment modalities in glaucoma in the future. While these therapeutics hold promise, further research is needed to optimize their efficacy, safety, formulation, and patient selection in clinic.

In addition, the emergence of a multitude of non-invasive sustained-release anti-glaucoma therapeutic modalities showcases the significant innovation in glaucoma treatment options and potential in the field of ocular pharmacology. Currently, the prostaglandin analogs-based delivery approaches are being intensively developed, among which the bimatoprost implant (Durysta) has been approved by the FDA and many other delivery devices are currently in Phase 2 and Phase 3 clinical trials, such as travoprost implant (iDose). From the available evidence, those glaucoma drug delivery implants have exhibited a great potential to optimize the effectiveness of therapies, increase patient compliance, and eliminate the issues related to the traditional daily eye drops (Miller and Eaton, 2021). Hence, the application of advanced drug delivery technologies to the anti-fibrotic agents discussed in this paper can further enhance their therapeutic efficacy, and will be a growing area of research to achieve greater clinical benefits in decreasing TM fibrosis and in improving the prognosis of glaucoma patients ultimately.

## Author contributions

MQ wrote the manuscript. CY led the study and wrote the manuscript.

## Author statement

We confirm that all authors have approved the manuscript and agree with its submission to European Journal of Pharmacology.

We confirm that neither the manuscript nor any parts of its content are currently under consideration or published in another journal.

## Declaration of competing interest

The authors declare that they have no conflict of interest.

## Data Availability

No data was used for the research described in the article.
